# Moving Forward Moving Backward: Directional Sorting of Chemotactic Cells due to Size and Adhesion Differences

**DOI:** 10.1371/journal.pcbi.0020056

**Published:** 2006-06-09

**Authors:** Jos Käfer, Paulien Hogeweg, Athanasius F. M Marée

**Affiliations:** Theoretical Biology and Bioinformatics, Utrecht University, Utrecht, Netherlands; Princeton University, United States of America

## Abstract

Differential movement of individual cells within tissues is an important yet poorly understood process in biological development. Here we present a computational study of cell sorting caused by a combination of cell adhesion and chemotaxis, where we assume that all cells respond equally to the chemotactic signal. To capture in our model mesoscopic properties of biological cells, such as their size and deformability, we use the Cellular Potts Model, a multiscale, cell-based Monte Carlo model. We demonstrate a rich array of cell-sorting phenomena, which depend on a combination of mescoscopic cell properties and tissue level constraints. Under the conditions studied, cell sorting is a fast process, which scales linearly with tissue size. We demonstrate the occurrence of “absolute negative mobility”, which means that cells may move in the direction opposite to the applied force (here chemotaxis). Moreover, during the sorting, cells may even reverse the direction of motion. Another interesting phenomenon is “minority sorting”, where the direction of movement does not depend on cell type, but on the frequency of the cell type in the tissue. A special case is the cAMP-wave-driven chemotaxis of *Dictyostelium* cells, which generates pressure waves that guide the sorting. The mechanisms we describe can easily be overlooked in studies of differential cell movement, hence certain experimental observations may be misinterpreted.

## Synopsis

The movements of biological cells during the development of an organism depend on the physical characteristics of these cells. Genetic regulation of (developmental) cell movements, in order to have an effect, must operate through or in accordance with these physical properties. With this framework in mind, the authors use a computational model to investigate the interaction of two important phenomena, namely differential adhesion and chemotaxis. Authors Käfer, Hogeweg, and Marée show that this interaction leads to fast cell sorting, a process during which the cells actually move in a specific direction, which can even be opposite to the direction of chemotaxis. The direction of motion does not only depend on intrinsic cell properties (such as cell size or the strength of the homotypic and heterotypic bonds) but also on the pattern formation that takes place during the sorting, as well as on general tissue properties. For example, both the formation of cell clusters and the level of confinement of the tissue can completely turn around the direction of sorting. The authors demonstrate that to fully understand cell tissue dynamics, it is essential to take into account mesoscopic cell properties, such as size, shape, and deformability. Because physically driven processes can profoundly influence the movement of biological cells, this should not be neglected in explanations for observed cell movement patterns.

## Introduction

The form of a multicellular organism is established by changing cell positions, configurations, and shapes. All these dynamics, orchestrated by cell differentiation and gene regulation, are mediated by basic physical processes such as cell adhesion, mechanical deformations, pressures within tissues, etc. [1,2]. Not only are these physical processes under genetic control, often they can feed back, through mechanotransduction cascades, on the gene regulation itself [3,4]. Therefore, it is essential to understand how the physical characteristics of the cells influence the cell dynamics, if we want to determine how gene regulation steers the development of an organism. Within this context, we focus on how cell adhesion influences the movement of chemotactic cells.

The classical experiments of Steinberg [5] show that dissociated cells reaggregate and sort out due to differential adhesion. Cells cohere to one another, or adhere to substrates, although often the term *adhesion* is used for both. Important for cell–cell adhesion are membrane proteins of the cadherin family, which are abundantly present in animal tissues, with their expression levels being tightly regulated [6,7]. Foty and Steinberg have shown that the surface tension of a tissue correlates with the number of surface cadherin molecules per cell, and that differences in surface tension (caused by different expression levels) are sufficient to generate cell sorting [8]. However, if the sole driving force for cell rearrangement and cell sorting were differential adhesion, then the sorting would depend on the fluctuations of cell movement, causing this process to be nondirectional and, as we will see, too slow to be of general importance for morphogenesis.

Chemotaxis has been found to be essential during development of diverse organisms and tissues. In the cellular slime mould *Dictyostelium discoideum,* chemotaxis towards cAMP guides the complete morphogenesis: from single cells to slugs, to fruiting bodies [9]. More recently the role of chemotaxis in the development of vertebrates and invertebrates has received attention. These developmental chemotactic systems are associated with FGFs [10,11] or with the *slit* gene family [12], for example, which are both widely present in vertebrates and invertebrates. Furthermore, in cancer metastasis, chemotaxis plays an important role [13] in association with differential adhesion [14].

Directional cell sorting is the process in which not only do cells sort out, but also each cell type ends up at a specific location or relative position. This is an important feature of *Dictyostelium* development: when aggregating, the amoebae differentiate in a random manner into the two major cell types involved in the morphogenesis. After the cell sorting, the prestalk cells occupy the anterior third of a migrating cell mass, while the prespore cells occupy the posterior part. This directional sorting cannot be due to differences in cell adhesion only, since differential adhesion does not supply a directional cue. Alternatively, it has been suggested that differences in chemotactic responsiveness between the cell types could determine this separation. However, it has become clear that both chemotaxis and differential adhesion are necessary for the process, but the relative importance of each process (or the possibility of yet another mechanism for directional cell sorting) is still the subject of debate [15–18].

Savill and Hogeweg [19] and Marée et al. [20] showed that differential adhesion combined with a chemotactic response, which is the same for all cells, is sufficient to cause directional cell sorting, offering thus a minimal set of requirements for the process in *Dictyostelium*. The mechanism has recently been verified experimentally [21]. Inspired by this case, our study considers homogeneous chemotactic responses to analyse the effects caused by differences in cell adhesion and cell size. Our results indicate that these cell properties do not uniquely determine the outcome of the sorting, but depend on the conditions in which cells find themselves: we will show that the fraction of each cell type in the tissue, the level of confinement of the tissue, and the manner in which the chemoattractant is distributed can completely turn around the outcome of the sorting.

We use a two-scale mesoscopic lattice-based model, introduced by Glazer and Granier [22,23], which has become known as the Cellular Potts Model (CPM). In this model formalism, cells have certain basic characteristics of biological cells: they possess a deformable boundary and may suffer small volume changes. The model is spatially explicit, with each cell consisting of multiple lattice sites. Real cells are highly dissipative objects, for which it holds that viscosity dominates inertia [24]. We therefore describe cell motion in terms of local energy gradients rather than through equations of motion in terms of explicit forces. The dynamics are based on the free energy minimisation principle [25,26], and generated by means of Monte Carlo simulations using the Metropolis algorithm [27]. Effectively, this means that cell motion comes about from the overall minimisation of the energy of deformation and stretching of the membrane through stochastic fluctuations, in which the global and local forces upon a cell are resolved [24]. The formalism has given the first realistic in silico description of cell sorting [22,23], and has also proven powerful for modelling biological morphogenesis [19,28,29].

In the model, a total energy cost is associated with the cell shapes and volumes, and fluctuations of the membrane permit the cell to explore its neighbourhood. The algorithm to generate the cell dynamics consists of randomly choosing a lattice site, calculating what the energy cost would be if a randomly chosen neighbouring site would extend itself into this site, and comparing this with the original energy cost. The probability of accepting the extension of the neighbouring cell (or medium) depends on the difference in the energy costs, with cell extensions that reduce it being accepted with higher probability. In this way, the cell shape is updated locally. These local updates are only weakly correlated with updates elsewhere in the same cell; this allows for cell deformations, as in real cells. For example, cells can be squeezed or can slide past one another.

In this study, the main driving force of all cells is chemotaxis. (We will look at two cases, the first being a constant chemoattractant gradient, the second a periodic chemoattractant wave). Chemotaxis itself is in both cases described by biasing the direction of cell extensions towards higher concentrations of the chemoattractant [19]:

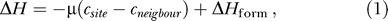
where Δ*H* is the energy cost difference for a given extension attempt, *μ* is the strength of the chemotaxis, and *c* is the concentration of a chemoattractant at a lattice site. Δ*H*_form_ is the energy cost difference that arises by changing the cell shapes by one site. The calculation of Δ*H*_form_ and the algorithm for calculating cell extension probabilities constitute the core of the CPM [22,23]. The CPM has its origins in physics, where it is used to describe foams [30], as an extension of the original Potts model (a generalised form of the Ising model in magnetism). To describe biological cells, Δ*H*_form_ is derived from the cell topology by calculating the total energy *H* of all cells *σ* before and after the attempted extension:


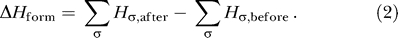


The energy cost *H_σ_* associated with the shape and volume of cell *σ* is given by


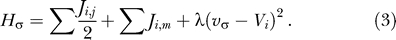


Here, *J_i,j_, J_i,m_* are the surface energies per boundary site between a site of cell *σ* and an adjoining site of a neighbouring cell or the medium, where *i* is the cell type of cell *σ*, *j* the cell type of the neighbouring cell, and *m* the medium. The surface energies are summed over all boundary sites of a cell, respectively, with the neighbouring cells and with the medium. The last term describes the volume constraint; *v_σ_* is the cell volume, *V_i_* the target volume of its cell type, and *λ* an “inelasticity” constant (see below). During one Monte Carlo time step (MCS), each site will be considered for a state change once, in a random order. The state of a lattice site is changed to the state of the randomly chosen neighbour with probability

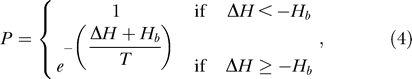
where *H_b_* represents a yield energy (the resistance to deform), and *T* is the “simulation temperature”, representing the membrane fluctuation amplitude of cells. The Monte Carlo algorithm, depending on the *T,* allows cells to explore ergodically the energy landscape, so that the whole tissue can evolve towards global energy minima.

Within this Hamiltonian-based formalism, local forces on the cell membrane are described implicitly. For an isolated cell, the positive energy associated with each membrane site will force the cell to minimise its boundary: it will become round and shrink. Shrinkage is counterbalanced by the volume conservation term in the Hamiltonian. Deviations from the target volume cause pressure changes in the cell, since pressure *p* is the conjugate variable to our volume variable *v_σ_*:


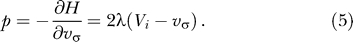


In this way, pressure will be constant throughout one cell.

In cell aggregates containing different cell types, the surface energies *J_i,j_* between a cell and its neighbours determine the cell shape and final configuration. Dynamics which are driven by energy minimisation basically consist of substituting high-energy bonds (i.e., which have a low adhesive affinity) with bonds of lower energy (i.e., which have a high adhesive affinity). In this study we focus on the interaction between two cell types, which we coined the dark type *(d)* and the light type *(l),* after how we depict them in the simulations. The surface tension between the two cell types is given by [23]

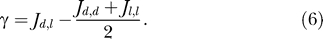
Negative surface tensions indicate that substituting two homotypic (one *l,l* and one *d,d* bond) by two heterotypic bonds is energetically favourable. Therefore, if *γ* is negative, cells tend to intermingle, creating checkerboard-like patterns, whereas with positive *γ* cells form homotypic clumps. Higher adhesions energetically favour having a common boundary, therefore low *J*'s (low costs) indicate strong adhesion. Both the surface tension between the cell types and those between each cell type and the medium determine whether overall the cells cluster together. Surface tensions with the medium were always chosen in such a way that two cells of different types adhere to one another, while together minimising their contact area with the medium, i.e., that all cells form a single clump.

This formalism enables us to represent the volume/pressure relationships, the viscoelastic properties, and the cytoskeleton-driven membrane fluctuations of biological cells in a straightforward way, as described in the following paragraph. The description of the pressure corresponds to a situation in which an infinitely fast redistribution of intracellular pressures occurs, i.e., pressure differences only occur between cells. This is a good description, because the volume changes in biological cells are not due to compression of the cytoplasm (the fluid inside cells is effectively incompressible), but due to the flow of water through the semipermeable cell membrane. The external pressure on the cell is actually counterbalanced by osmotic pressure inside the cell, as has been shown experimentally for *Paramecium* [31]. Pressure changes outside the cell therefore require corresponding changes in the osmotic pressure in the cell, since large pressure differences would disrupt the membrane. Osmotic pressure can change by a water flow into or out of the cell, the process described by the volume conservation term. Cells can also respond to pressure differences by actively changing their osmolarity in order to maintain their original volume [31].

Within our modelling framework, this would correspond to changing the target volume in response to large changes in pressure; in this study, however, such regulation is not considered. The parameter *λ* describes the volume conservation caused by the viscoelastic properties of the cell. For larger *λ,* cells more stringently keep their target volume by being less flexible to volume changes, and therefore movement becomes more difficult. Guilak et al. [32] and Trickey et al. [33] studied such viscoelastic properties of articular chondrocytes, showing that cells are indeed compressible under mechanical force (chondrocytes have a Poisson's ratio of 0.36, i.e., significantly below 0.5, meaning that under pressure the cells are not able to keep their volume). Using alterations in the osmotic environment to determine the limits of the volume changes, they found that cells can undergo more than three-fold volume changes until lysis. Here, we keep variations in cell volume due to the tissue dynamics limited to at most 10% (a conservative approach, considering the data of [32] or [31], who observed variations up to 20%).

We would like to point out, however, that when variations in volume are kept within an even smaller range (by increasing *λ*), all results remain qualitatively the same, only the timescales of sorting become longer, due to the above-mentioned decrease in movement. The value of the simulation temperature *T* sets the amplitude of random fluctuations of the cell boundary, and this represents the active, cytoskeleton-driven membrane fluctuations, which allow cells to actively explore their neighbourhood. Mombach et al. [34] have shown that the effects of the drug cytochalasin-B (a suppressor of membrane ruffling) on biological cell dynamics, can indeed be very well described within the CPM as a reduction of *T*. The yield *H_b_* describes the fact that cell membranes display a certain level of resistance to deform, mainly due to the internal cytoskeleton architecture [35].

Here, we explicitly study the behaviour that results from the entanglement of chemotaxis and differential adhesion, instead of focusing on chemotaxis or differential adhesion separately. We analyse what basic physical features define the outcome of the cell sorting, and how these dynamics can be understood in terms of these features. Surprisingly, the outcome turns out not to be defined by cell properties only, but depends in an essential way on tissue properties, such as the level of confinement of the tissue, which feed back to the sorting. We explore and unravel these dependencies, and suggest experiments to test the proposed mechanisms.

## Results

In this section we first discuss cell sorting in a static chemotactic gradient: it is essential to understand this simpler case before generalising the results to the more complicated periodic chemotactic waves that operate in D. discoideum. For the static gradient we first demonstrate that chemotaxis speeds up cell sorting by orders of magnitude. To understand this, we next analyse in detail cell sorting in a confined cell mass (where we introduce a barrier, a “wall” that the cells cannot pass), showing that both cell size and cell adhesion determine speed and direction of cell movement. Understanding the mechanisms involved allows us to predict the very different cell-sorting phenomena in freely moving cell masses and the influence of other tissue level properties, not only in a static chemotactic gradient, but also in the more complicated case of a dynamically changing chemotactic gradient, as in *Dictyostelium,* which is discussed in the last section.

We should note that the confined cell mass as well as the freely moving cell mass studied here, are extreme cases relative to what occurs in organisms. In most developmental processes, some type of confinement will play a role, although not in the form of the incompressible wall in the direction of chemotaxis, which we study. Instead it will consist of other tissues that are relatively inert. Likewise, even in *Dictyostelium* slugs, which are an extreme example of a freely moving cell mass, the migrating slug is to a certain level confined by the surrounding slime layer, and the tip of the slug that has to be pushed forward.

### Static Chemotactic Gradient

#### Chemotaxis accelerates cell sorting.

Here we analyse the dynamics of a tissue spread over a two-dimensional (2-D) plane in which all the cells have the same chemotactic response, which, as shown in Equation 1, represents the tendency of a cell to move along a local chemical gradient. Consequently, any observed cell rearrangement or (directional) cell sorting can only be linked to differences in size and/or differential adhesion. We start with a small fraction of one cell type (the “dark” cells), to ensure that initially most dark cells are isolated from one another, so we can study the process of cell sorting ab initio. Later we further explore the consequences of the relative population sizes. We will refer to directions relative to the direction of chemotaxis; “forward” motion is movement in the chemotactic direction, “backward” is in the opposite direction.

Differential adhesion alone is sufficient to drive cell sorting [22,23]. Yet, the process slows down logarithmically [36], so the timescales involved for complete sorting do not correspond to the biological timescales observed in most morphogenetic processes. Apparently other processes are involved as well, which significantly speed up the process. Chemotaxis is such a process that has the capacity to accelerate cell sorting. This is demonstrated by the following simulations, where we compare a purely adhesion-driven sorting to the effect of adding different levels of chemotactic responses. We use periodic boundary conditions; the difference can therefore not be attributed to a “heaping up” of one cell type at one side.

**Figure 1:**
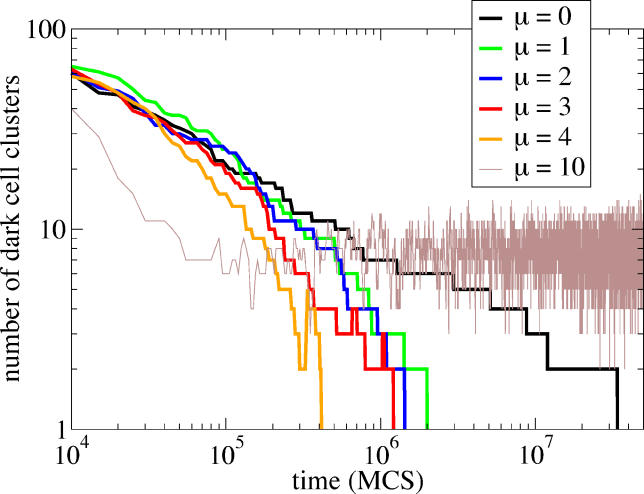
Rates of Cell Sorting for Different Chemotactic Strengths Influenced by non-specific chemotaxis, cell sorting can become 10–100× faster than for pure differential adhesion. Parameters are *J_l,l_* = 5, *J_d,l_* = 9, *J_d,d_* = 8 (yielding *γ* = 2.5), *V_l_* = *V_d_* = 30, *T* = 6, *λ* = 12, and *H_b_* = 0.8.

Figure 1 shows the general effect of chemotaxis on the rate of cell sorting. For a densely packed cell mass, we determined the reduction in the number of cell clusters due to the fusion of small ones into larger ones, which is a good measure of the cell sorting. Without chemotaxis, cell sorting only occurs if the surface tension (Equation 6) between the cell types is positive. But even then, complete sorting is slow: without chemotaxis (*μ* = 0), the decrease in the number of clusters over time gives an approximately straight line on a log–log scale, which means that the sorting slows down as a power-law, i.e., it slows down logarithmically. This is due to the fact that increasingly larger clusters are formed, which not only displace much more slowly, but are also at larger distances from one another. Thus, while complete cell sorting corresponds to the minimal energy configuration, reaching this state takes a very long time. Increasing the simulation temperature, *T,* does increase the rate of sorting (unpublished data). The sorting, however, always slows down logarithmically, which means that higher temperatures cannot eliminate the large timescale involved in the final stages of the sorting. Moreover, increase of tissue size is accompanied by a more than linear increase in sorting time, while the extent to which high temperatures can speed up sorting is limited, due to the existence of critical temperatures for cell integrity and stable cell cluster formation [23].

Cell sorting, however, can be two orders of magnitude faster when the cells move chemotactically (Equation 6). Chemotaxis is not equivalent to increasing the effective temperature of the simulation, because it favours extensions in one direction while at the same time inhibiting in the other, therewith not changing the ratio between attempted and accepted cell extensions. Instead, the sorting is caused by the fact that (clusters of) cells with different surface energies move at different speeds (even though the chemotactic response is equal for both cell types), therewith continuously causing medium-scale tissue rearrangements, which strongly reduces the collision time between clusters. In contrast to the process driven by differential adhesion alone, the sorting only slows down exponentially, and scales linearly with tissue size (since it depends on relative speed differences). When the chemotaxis is too strong (e.g., *μ* = 10), the flow becomes highly turbulent, and clusters are continuously disrupted, preventing full sorting. Realising that chemotaxis has such a large impact on sorting by differential adhesion (which cannot be mimicked by increasing the simulation temperature, i.e., the mobility of the cells), we first want to unravel how both cell properties interact to cause (directional) cell sorting. We then determine the role of specific tissue properties in the process.

#### Chemotaxis can generate a pressure gradient.

**Figure 2:**
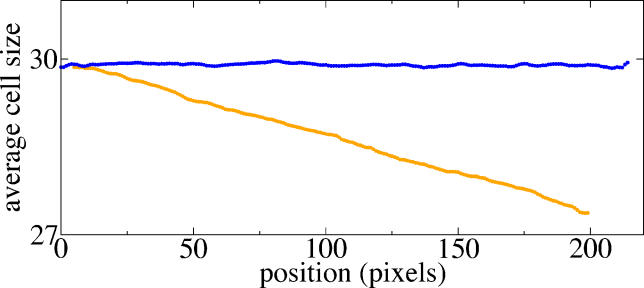
Average Cell Sizes in a Homogeneous Cell Mass Moving Chemotactically to the Right Motion is either unrestricted (blue line), or confined at position 200 (orange line). When motion is confined, a pressure gradient forms that counteracts the chemotaxis. Cell sizes are the average for each column during 50 MCS, sampled after 15000 MCS. *J_l,l_* = *J_l,m_* = 5, *μ* = 2, no dark cells; other parameters as in Figure 1.

Whether chemotactically moving cell masses are confined or move freely determines their direction of cell sorting (see below). This is due to the fact that in confined cell masses, unlike in freely moving cell masses, the chemotactic motion of the cells gives rise to a volume gradient, as shown in Figure 2. For our choice of cell inelasticity, *λ,* the largest volume differences are approximately 10%. The volume gradient forms quickly, after which it remains approximately stationary. The volume differences are due to the pressure on the cells, as described in Equation 5; so one can see the volume gradient as a direct consequence of the pressure gradient. When the gradient is stationary, the spatial derivative of the pressure (i.e., the slope in the volume times 2*λ,* see Equation 5) is counterbalancing an imposed force on the cell, in this case the force generated by the chemotaxis. In the freely moving cell mass, however, the forces do not have to be balanced, as the cell mass simply moves forward, and the cells tend to maintain their target volume.

#### A pressure gradient causes size-based cell segregation.

**Figure 3:**
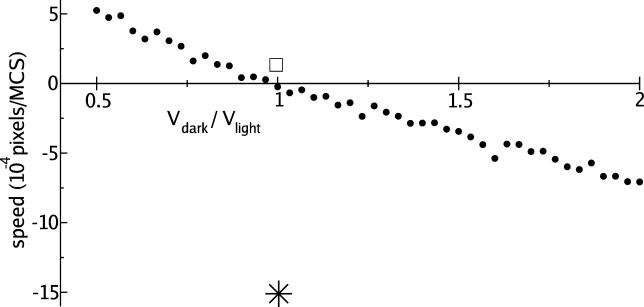
Size-Based Cell Sorting in a Confined Cell Mass The graph shows the rate by which the mean position of the dark cells shifts, relative to the mean position of the light cells, averaged over 50,000 MCS. Positive values indicate forward movement of the dark cells. The graph shows that small cells move forward, while large cells move backward. For both *V_dark_/V_light_* < 1 and *V_dark_/V_light_* > 1, the graph is approximately linear, with *y* = 9.99 · 10^−4^(1 − *x*), and *y* = 7.18 · 10^−4^ (1 − *x*), respectively (for both line segments, the correlation coefficient is 0.990). *J_l,l_* = *J_d,l_* = *J_d,d_* = 5, *J_d,m_* = 4, *J_l,m_* = 5, *T* = 6, *λ* = 12, *μ* = 2, *H_b_* = 0.8, and *V_l_* = 60. The square and star indicate the effective volume ratios versus shifting rates for Figure 5A–5C and Figure 5D–5F, respectively.

**Figure 4:**
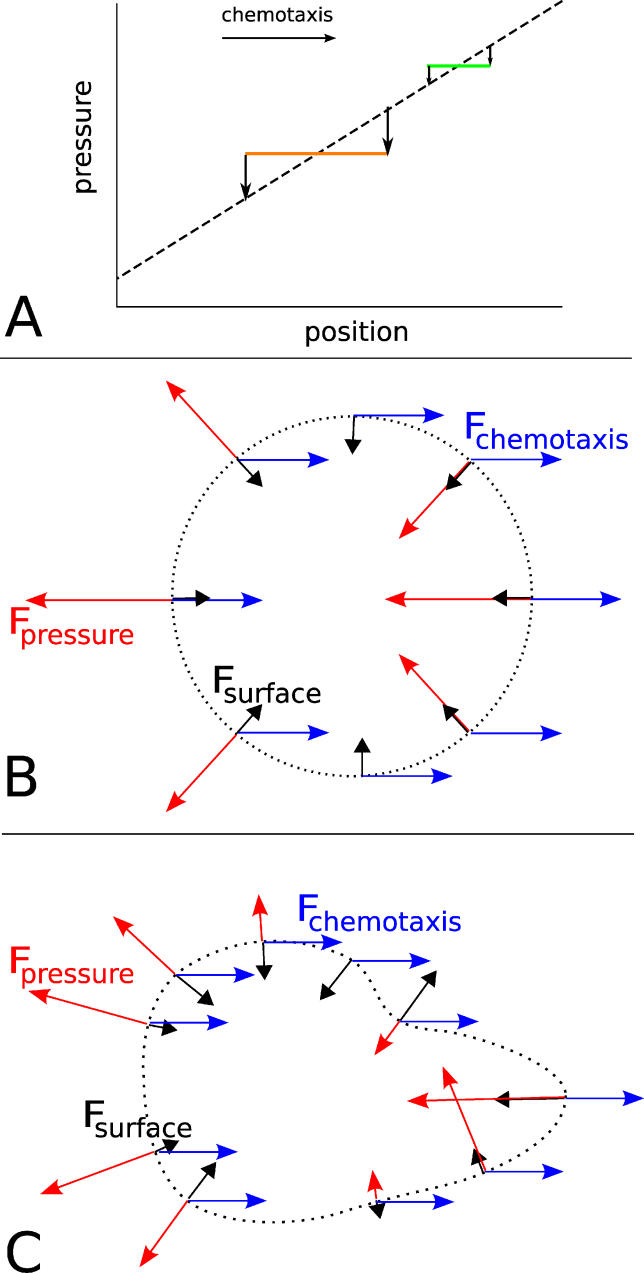
Schematic Representation of the Effect of Pressure and Chemotaxis on Cell Shape within a Confined Cell Mass (A) A small (green) and a large (orange) cell within a pressure gradient. The dashed line represents the average pressure in the overall cell mass (cf. Figure 2). Throughout each cell, however, the pressure is constant. The vertical arrows represent the pressure-driven tendency to extend into cells with a lower pressure, and therewith the tendency of each cell to move backward. At the extremities of a large cell, the pressure differences are larger than at the extremities of a small cell; therefore a large cell moves backward faster. (B) Orientation and magnitude of the forces exerted due to pressure and chemotaxis. Forces act upon the cell membrane (dotted circle). The magnitude and direction of the chemotaxis (blue arrows) is constant along the boundary. The forces due to pressure differences (red arrows) vary in magnitude and direction: large and inwards at the front, large and outwards at the back, and small in the centre. Cells tend to round up due to the surface forces (black arrows), which are determined by the shape of the cell, and limit the level of deformation. The latter forces are directed perpendicularly to the membrane, with sign and magnitude depending on the curvature. (C) A deformed cell (dotted line). The pressure (red arrows) and surface (black arrows) forces are again perpendicular to the membrane. For the surface forces, the magnitude is proportional to the curvature, so the force is directed towards the concave side. The chemotactic forces (blue arrows) remain the same. Note that the surface and pressure forces are not defined as such in the model; they arise from the minimisation of Equation 3. The chemotactic force is simply a consequence of Equation 1.

Once such a pressure gradient has been established through the tissue, size differences between cells can be shown to be sufficient to cause directional cell sorting. Figure 3 shows the effect of size differences on the speed and direction of movement. Depicted is the relative rate by which the dark cells, the minority, displace, as a function of how much their target volume, *V*_dark_, deviates from the fixed target volume of the majority of cells, *V*_light_. The speed of cell sorting depends approximately linearly on the ratio of the target volumes of both cell types, in which the largest cells always move backward, i.e., opposite to the direction of chemotaxis. Video S1 illustrates this behaviour. The contra-chemotactic movement of larger cells is due to an effective backward force caused by the pressure gradient. As depicted schematically in Figure 4 and described hereafter, it is important to realise that the forces on the cell act on a subcellular scale. There is a pressure gradient throughout the whole tissue, and if cells were point-like objects, there would be the same backward force everywhere in the tissue, exactly counterbalancing the chemotaxis. However, cells have a mesoscale structure, while within the whole cell the pressure is constant. This implies that if the neighbouring cell is under higher pressure, there is a force directed inwards, while if the pressure is lower, the force is directed outwards. At the front (defined by the direction of chemotaxis), neighbouring cells are overall under larger pressure, while at the rear, the pressure is lower. Consequently, cells tend to extend at the rear and retract at the front, i.e., within a pressure gradient the cells tend to move backward. If all cells are equal, this leads to a small drop in the pressure gradient, until the backward motion is counterbalanced by the chemotaxis. Larger cells, however, span a wider distance within the pressure gradient, and the pressure differences at both extremes of the cell are therefore larger (see Figure 4A). Consequently, both at the front and the rear the backward force is stronger for the large cells, causing movement towards lower pressure, which is in the opposite direction as the chemotaxis (see also Video S2 and the section about the Robustness of the Formalism).

**Figure 5:**
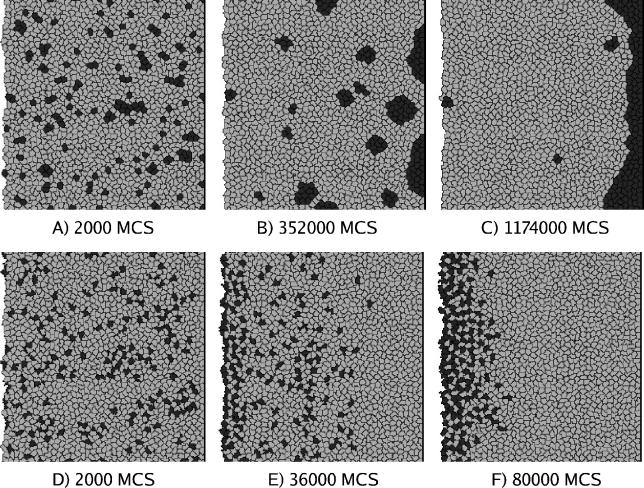
Snapshots of Two Typical Runs That Show Adhesion-Based Cell Sorting The upper panels show directional cell sorting of the cells with high surface energy towards the front (in the direction of the chemotaxis), while in the lower panels the cells, which have low surface energy, sort to the back. (A–C) *J_l,l_* = 5, *J_d,l_* = 9, *J_d,d_* = 8 (*γ* = 2.5). (D–F) *J_l,l_* = 5, *J_d,l_* = 3, *J_d,d_* = 3 (*γ* = −1). Other parameters as in Figure 1.

There are two important things to note here. First, not only large cells, but all cells, have the tendency to be pushed backward at their extremities, where the force generated by the pressure dominates over the chemotaxis (see Figure 4B). In a homogeneous cell population, however, this is being compensated by extra extensions in the more central part of the cell, because overall the cells are not being displaced. Since continuously different parts of the cell extend and contract, the dynamics do not reach equilibrium, and the cells never stop pushing each other away. This means that the stable pressure gradient observed on the macroscale is sustained in a highly dynamical way, i.e., like the Red Queen, the cells have to move continuously to stay at the same place: *“A slow sort of country!” said the Queen. “Now, here, you see, it takes all the running you can do, to keep in the same place. If you want to get somewhere else, you must run at least twice as fast as that!”* [37]. Second, the viscosity of cells does not play a role in this process, since all cells can be approximated as being highly viscous. What is important, therefore, is the amount of force that is required to deform the cells, because more flexible cells are pushed backward more easily by the other cells. This flexibility is in large part determined by the adhesion properties of the cell, as we will discuss in the next section.

### Differential Adhesion

#### Anisotropic pressure environment in a static chemotactic gradient.

Figure 5 shows two examples of differential adhesion causing directional cell sorting. The snapshots show a confined cell mass with chemotaxis to the right. In Figure 5A–5C, the high-surface-energy dark cells move forward, while in Figure 5D–5F the low-surface-energy dark cells move backward, against the direction of chemotaxis. Given the fact that cells with a higher surface energy (but the same target volume) overall are smaller (which follows from Equation 3), a first thought would be that directional cell sorting is caused by this volume difference, in the way described above. However, the volume differences turn out to be far too small to explain such rapid sorting. (See the square and the star in Figure 3; the shifting rates are, respectively, 33 and 701 times faster than would be expected from the volume differences only.)

Therefore, the main mechanism must be directly linked to the surface energy itself. In the quasi-stationary situation, when on the macroscale the pressure gradient is counterbalancing the chemotaxis, the mean cell shape is actually anisotropic, due to the spatial imbalances within each cell between extending and retracting. This is due to the fact that on the subcellular scale chemotaxis and pressure act spatially different. The chemotactic force is the same along the whole cell, and is always directed forward. In contrast, the force generated by the pressure differences varies strongly along the boundary of the cell, and is always directed perpendicular to the cell boundary (i.e., the force is large and directed backward at the extremities of the cell, but becomes smaller closer to the centre, being directed inwards towards the front and outwards towards the rear). This results in cells that have the tendency to move forward due to chemotaxis, but are squeezed by the high pressure of their neighbours at the front, and widened by the low pressure of their neighbours at the back (see Figure 4B and 4C). Consequently, a cell in general has a drop-like shape, wide in the back and smaller towards the front. Cells with a lower surface energy are more flexible, because the “cost” of having non-optimal shapes is lower (since non-optimal shapes have a higher perimeter/area ratio and thus higher surface energies). Consequently, their shapes are more anisotropic, or, in other words, they are effectively squeezed backward by the rounder, more rigid, high-surface-energy cells. The anisotropic shape of the cells can be measured in the simulations by analysing the mean volume distribution of a cell over time, relative to its centre of mass. This distribution is always skewed in the direction of chemotaxis. The skewness or third moment (which is defined as 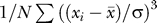 , where the sum is taken over all sites that are part of the cell, and *σ* is the distribution's standard deviation) is a good measure to quantify this shape as anisotropy. Positive values (assuming chemotaxis to the right) indicate that cells are thinner and more elongated in the direction of chemotaxis, and are thicker and shorter in the opposite direction. Its value is higher when the cells are easier to deform, i.e., for cells with a lower effective surface energy, or alternatively, for lower values of *λ*. For example, when *J_l,l_* = 7, *J_d,d_* = 11, *J_d,l_* = 3, *λ* = 2, with all other parameters as in Figure 3, the mean skewness of the dark cells in the *x*-direction is 0.31. In the *y*-direction, perpendicular to the chemotaxis, the mean skewness is always zero, as is to be expected.

Thus, an individual cell with a lower surface energy undergoes a shape change in such a way that it generates a backward motion. Yet, the adhesion properties are important for the surface energy, and they depend on the interactions that take place between the cells, and on the way these interactions feed back. Moreover, during the cell sorting, increasingly larger clusters of cells are formed, which not only change the local neighbourhood of individual cells, but also add another layer, determining the outcome of the directional cell sorting. The direction of motion, therefore, cannot be studied as if it is independent from the cell sorting.

#### Effective surface energy determines the direction of sorting.

**Figure 6:**
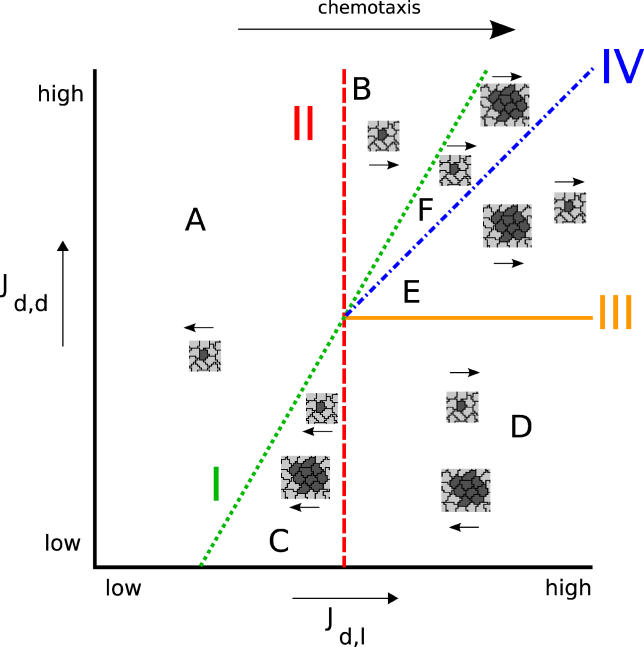
An Overview of Dependency of the Direction of Cell Sorting, for Both Single Cells and Small Clusters, on the Specific Combinations of Surface Energies, in a Confined Cell Mass *J_l,l_* is fixed; where the lines I–IV intersect, *J_l,l_* = *J_d,l_* = *J_d,d_*. Line I corresponds to *γ* = 0 (Equation 6); line II to *J_d,l_* = *J_l,l_;* line III to *J_d,d_* = *J_l,l_;* and line IV to *J_d,d_* = *J_d,l_*. Arrows indicate the direction of sorting of the dark cell type, when chemotaxis directs cells to the right and movement is constrained. The arrows solely indicate the direction of sorting; quantitative differences between single cells and clusters are not shown. For movies of the cell behaviour in each region, see Videos S3–S8.

Figure 6 depicts qualitatively the behaviour of dark cells for various adhesion strengths. For movies of the cell behaviour in each region, see Videos S3–S8. The surface energy depends on properties of both cell types. To determine the “effective surface energy” (i.e., the contribution of the first two terms in Equation 3 for a specific cell at a specific moment in time), we need to know the neighbours of the cell, the length of the interface with each neighbour, and the per-bond surface energy *(J_i,j_)* with that neighbour.

**Figure 7:**
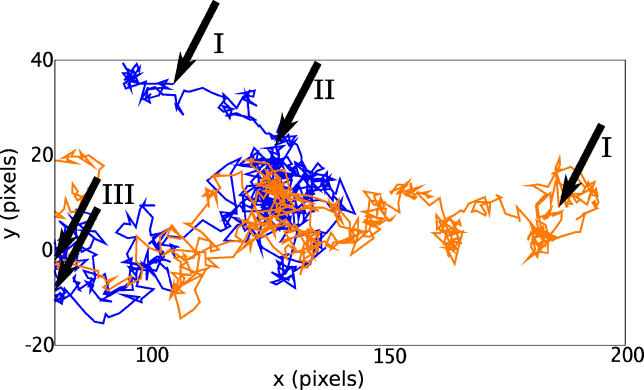
Cell Tracking of Two Individual Dark Cells in a Cell Mass Chemotactically Moving to the Right The arrows I indicate the initial positions of the cells, the arrows III the final positions. The cell represented by the orange track almost immediately becomes part of a cluster, and slowly moves backward, while the cell represented by the blue track initially moves forward as a single cell, but later on joins a cluster (arrow II), and then moves backward. *J_l,l_* = 5, *J_d,l_* = 6, and J*_d,d_* = 3; other parameters are as in Figure 5. This behaviour is illustrated by Video S6.

We find that in a confined cell mass, the dark cells move forward if their average effective surface energy is higher than the average effective surface energy of the light cells, otherwise they move backward (as is to be expected from the anisotropic pressure environment). The easiest situation to determine the direction of motion, is when the surface tension (Equation 6) is negative, while the dark cell density is sufficiently small (regions A and B in Figure 6). In this case, *J_d,d_* is of little importance, because dark cells do not form clusters: almost all dark cells border light cells, while the light cells predominantly maintain homotypic, light neighbours. The effective surface energy for the dark cells is therefore close to *J_d,l_,* while for the light cells it is close to *J_l,l_*. Now, if *J_d,l_* < *J_l,l_* (region A), the dark cells have the lowest effective surface energy, and consequently move backward; if *J_d,l_* > *J_l,l_* (region B), the dark cells move forward. When the surface tension is positive (to the right of the green line I), dark cells cluster together and *J_d,d_* starts to play a significant role. Now, if both *J_d,l_* and *J_d,d_* are smaller than *J_l,l_* (region C), dark cells move backward. Because *J_d,d_* < *J_d,l_* (the positive surface-tension requirement), the effective surface energy of dark cells within clusters is smaller, so clusters move faster backward than individual cells. A more evident example of this effect is seen in region D, where *J_d,l_* > *J_l,l_*, while *J_d,d_* < *J_l,l_*. Because *J_d,l_* > *J_l,l_,* single cells move forward. However, while the dark cells cluster together, *J_d,d_* becomes increasingly more important, which lowers the effective surface energy until the cluster becomes large enough to change its direction of motion and move backward (see Figure 7 and Video S6). In regions E and F of Figure 6, the dark cells always move forward, because both *J_d,l_* and *J_d,d_* are larger than *J_l,l_*. Clumps move slower than single cells in region E (where *J_d,d_* < *J_d,l_*), and faster in region F (where *J_d,d_* > *J_d,l_*), but the differences are small.

When within clusters the effective surface energy is lower than within the rest of the tissue, the pressure gradient within the cluster becomes less steep, compared with outside of the cluster. Consequently, large pressure differences appear at both extremities of the cluster, which strongly directs it backward, in the same way as large cells are pushed backward. This can further enhance the reversal of direction due to cluster formation, as is shown in Figure 7 and Video S6.

To summarise, the direction of sorting is not just determined by the homotypic and heterotypic per-bond surface energies, but also depends on mesoscale pattern formation, both because the patterns change the specific neighbours of cells, and because clusters as a whole respond differently to the pressure gradient. Moreover, properties of the tissue as a whole also have a strong impact on the outcome of directional cell sorting, which we will discuss in the next section.

### Tissue Properties

Until now we have focused on a specific set of tissue properties (confined cell mass, fixed unequal cell-type fractions, and fixed chemoattractant gradient), in order to be able to unravel the mechanisms underlying differential cell sorting. We now ask the question what are the consequences of these assumptions. The answers, it turns out, follow naturally from the above observations.

#### What happens in freely moving cell masses?

As shown in Figure 2, when the cell mass is not confined, no pressure gradient forms. Consequently, no pushing backward takes place. The cell mass steadily moves forward, leaving as the only variation to movement the degree of ease with which the forward dislocation occurs. This question is easy to answer: when the effective surface energy is low, cells are more flexible, and hence they can move faster. Thus, the lack of a pressure gradient creates an inverted picture from what was seen for freely moving cell masses: the relationship between effective surface energy and direction of cell sorting flips, which means that all arrows in Figure 6 reverse. For example, if *J_d,l_* < *J_l,l_* and the surface tension is negative (i.e., region A of Figure 6), the dark cells now sort to the front of the moving cell mass. To predict the outcome of a cell-sorting experiment, it is therefore essential to determine the level of confinement of the tissue, or better, the strength of the pressure gradients.

#### Does the relative amount of each cell type play a role?

As we have shown above, the direction of sorting cannot be determined without taking into account the relative amounts of each cell type, because the direction is determined by the effective surface energy, defined by the cell *and* by its neighbours. This is both important when the surface tension is negative, in which case all cells are predominantly surrounded by the most common cell type, and in the case of positive surface tensions, when clusters are formed, since the pulling on the clusters strongly depends on the curvature of the boundary, due to which it effectively only takes place for the minority cell type. An especially interesting case is when *J_d,d_* = *J_l,l_,* because in this case the direction of sorting depends on the amount of each cell type only: if the dark cells are in the minority (as is the case for Figure 6), they move within a confined cell mass in the chemotactic direction if *J_d,l_ > J_l,l_;* however, if more than 50% of the cells are dark, the directions reverse, and instead they move forward when *J_d,l_ < J_l,l_*. Because the direction of movement is determined by the relative amount of two otherwise similar cell types, we have coined this “minority sorting”.

#### Could a different chemotactic signal change the results?

When in a freely moving cell mass all cells exert the same chemotactic force, no pressure differences appear; the tissue simply displaces. However, when there are spatial differences in chemotactic strength, the situation immediately becomes very different: the cells with a stronger chemotaxis tend to move faster until this effect is compensated for by a pressure gradient, which, as before, will slow down the cells by creating backward force. Since the presence or absence of a pressure gradient has such a direct influence on the cell sorting, the outcome in a non-homogeneous chemotactic situation can be totally different from the homogeneous case. In this respect, the sorting that takes place in the cellular slime mould D. discoideum is particularly interesting, because of the spatio-temporal oscillations in the chemotactic process, caused by cAMP waves, which are therefore associated with pressure waves [38].

### Directional cell sorting in *Dictyostelium.*

The directional cell sorting, which takes place during the mound and early migration stages of the cellular slime mould D. discoideum [39], has been modelled with the CPM before [19,20,40]. *Dictyostelium* cells respond to a diffusible signal molecule, cAMP. Important for our study are two cellular responses to cAMP: wave-like cAMP relay by the cells (because of the excitable medium dynamics of cAMP production) and chemotaxis towards higher concentrations of cAMP. cAMP waves are followed by a refractory period, in which cells are non-responsive to cAMP (neither cAMP production nor chemotaxis). Due to the wave-like nature and refractoriness, cells are only chemotactically active during a short period, unlike the cases we studied above, causing the creation of complex pressure gradients.

#### A composite pressure gradient influences sorting.

**Figure 8:**
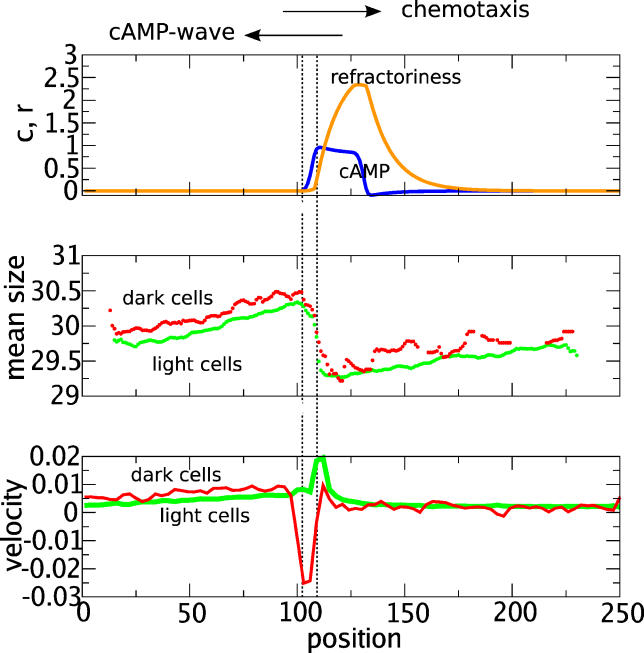
Volume Gradient and Cell Speed of a Freely Moving Cell Mass Chemotactically Responding to a Periodic cAMP Signal The cAMP wave travels from the right to the left (as shown in the top panel, where *c* is cAMP, blue line; and *r* refractoriness, orange line). Cells only move chemotactically between the dashed vertical lines, when cAMP > 0.05 and refractoriness < 0.2. Consequently, chemotaxis is always directed to the right. In the middle panel, red and green dots indicate the average size of, respectively, dark and light cells. The bottom panel shows the average speed of the dark and light cells (given by the red and green lines, respectively). Parameters are *J_l,l_* = 5, *J_d,l_* = 3, *J_d,d_* = 3 (*γ* = −1), *λ* = 5, *μ* = 200, *V* = 30, *T* = 6, and H_b_ = 0.8; cAMP dynamics are as described in [20], except that here the medium is also part of the excitable tissue, and that, throughout the tissue, excitability is 0.1. Average sizes were calculated as in Figure 2; average speeds were calculated by averaging the displacements of all cells within three-lattice-sites-wide strips over 100 intervals of 50 MCS, using a moving coordinate system linked to the cAMP wave.

Figure 8 shows the cAMP waves and refractoriness, volume gradient, and cell speed for a freely moving cell mass. (We do not yet use a specific “slug” shape.) Because cells are only chemotactically active during the cAMP wave (between the dashed vertical lines), a complex pressure wave forms. When the cells are active, their sudden strong chemotactic motion creates a steep pressure gradient in the opposite direction, but also a pressure gradient outside the chemotactically active part, oriented in same direction as the chemotaxis. Consequently, even before the arrival of the cAMP wave, cells are pulled forward by the chemotactically moving cells in front of them, while after the wave cells are pushed forward [19,29]. This can be seen in the bottom graph of Figure 8: due to the generated pressure wave, the speed of the cells that do not move chemotactically is still positive. (Note that if the movement of the cell mass were confined, there would be almost no pressure gradient outside the chemotactically active region, and therefore no forward movement, except by the chemotactically moving cells; unpublished data).

Within this setting, cell sorting due to differential adhesion is more complex, because the direction of motion can change sign in different parts of the cell mass, depending on the position of the cAMP wave. The bottom panel of Figure 8 shows that the more flexible dark cells move backward during the wave, but otherwise forward, which means that these cells only move in the direction of chemotaxis *when they are not chemotactically active*. This logically follows from the fact that, as discussed previously, the most flexible cells respond most strongly to the pressure gradient. The steep pressure gradient in the region of the cAMP wave leads to backward motion, the cells being pushed away by the light cells with a higher effective surface energy. In contrast, the shallow gradients outside the chemotactic region, with their opposite orientation, lead to forward motion, which is faster than for the light cells. Because the cAMP wave takes up such a small fraction of the time, the non-chemotactic motion turns out to determine the end result, and the dark cells sort to the front. Clearly, this outcome would have been different, when the spatio-temporal pressure distribution would have been different: when the region where the pressure is directed in the opposite direction would have been large, the dark cells would have sorted out to the back.

#### Sorting in *Dictyostelium* slugs.

**Figure 9:**
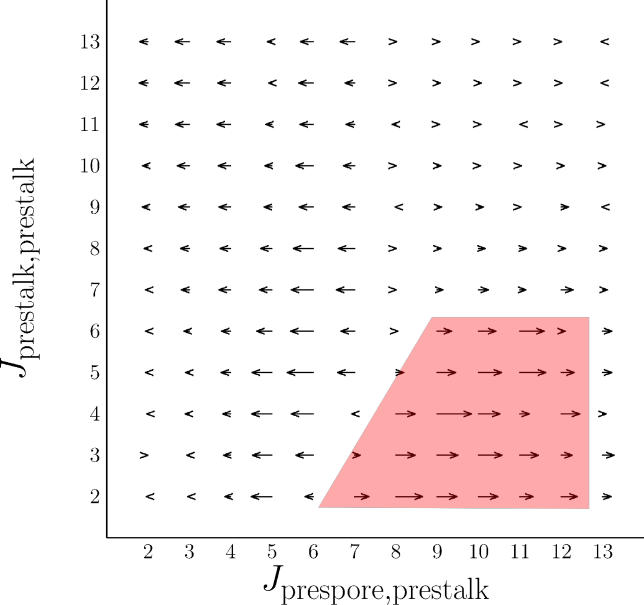
Direction of Cell Sorting of the Prestalk Cells in Simulated *Dictyostelium* Slugs Arrows pointed toward the right indicate movement of these cells to the anterior of the slug. Cell types are: *a* = pacemaker, *t* = prestalk, *p* = prespore, and *m* = medium. In all cases, *J_p,p_* = 11. The shaded area indicates fast-forward sorting of the prestalk cells, the left border of this area corresponding to γ = 0 (Equation 6). Parameters are *J_a,a_* = 3, *J_a,m_* = 7, *J_t,m_* = 8, *J_p,m_* = 11, *J_a,t_* = 6, *J_a,p_* = 9, *T* = 2, *λ* = 1, *H_b_* = 0.8, and *μ* = 200; cAMP dynamics are as described in [20].

The migrating cell mass of *Dictyostelium,* called a “slug”, consists of three major cell types. Prestalk cells occupy the anterior third of the cell mass, while the rest consists of prespore cells. Prestalk A cells in the tip of the slug act as pacemakers from which the cAMP waves originate, while prestalk O cells and prespore cells relay the signal. Figure 9 shows that the prestalk cells (minority) can only sort to the anterior if the surface tension is positive and *J_prestalk,prestalk_* is sufficiently smaller than *J_prespore,prespore_* (shaded area). This is actually due to the fact that in the tightly packed slug, cells push and pull each other: the outcome is determined outside the cAMP region. The arrows in Figure 9 neither correspond to the ones in Figure 6, nor to its inverse, for two reasons. First, because of the flipping of the pressure gradient, the outcome is a complex integration of the dynamics in the different zones. Second, the tip must be pushed forward by the moving cell mass. It therefore functions as a movable obstacle, causing the slug to be intermediate between a confined and a freely moving cell mass. Varying the strength of the surface energy with the medium, the inelasticity *(λ),* or the temperature *(T),* makes the slug more or less confined. This changes the shape of the pressure gradients, allowing the surface energy-sorting relationship to more or less resemble (or mirror) Figure 6. It illustrates the complexity of translating specific cell properties, such as its adhesion and cohesion, into expected global dynamics.

### Robustness of the Formalism

The CPM is a discrete, lattice-based formalism. To rule out the possibility of implementation-based biases, we performed a number of controls.

First, to rule out lattice effects, we ran our model on a three-dimensional (3-D) cubic lattice as well as on a 2-D hexagonal lattice. The directions and relative differences in speed were the same as in the presented 2-D model on a square lattice.

Second, in our model chemotaxis takes place along the whole cell boundary. Real cells might localise their chemotactic response to the front-most part of the cell. We tested if this would make any difference. Both when we restricted the chemotaxis to a fixed percentage of the cell boundary or to a fixed number of lattice sites, when a correct scaling was applied, the results did not change.

Third, we have verified the correctness of our explanation of the observed dynamics in terms of the generated pressure gradient, i.e., whether a different effect of the chemotaxis can be excluded. To test this, we generated a pressure gradient within the model by means of pushing a cell mass forward with constant speed (implemented by regularly shifting a confined boundary one column to the right and correcting for the reduced cell volumes). In this case, larger cells move faster in the direction in which the cells are being pushed (Video S2), which again corresponds to movement towards lower pressure. Note, however, that pushing the tissue generates a pressure gradient which is opposite to the confined-cell-mass case (Video S1), and therewith flips the direction of motion. The same kind of dynamics have also been found experimentally and predicted theoretically (on basically the same grounds) [41] for large bubbles in plug flow of 2-D foams, illustrating that this is a general property of systems built up from mesoscale deformable structures, such as cell tissues and foams. In the case of differential adhesion, like before, the cells with the lowest effective surface energy responded strongest to the pressure gradient.

Fourth, we checked if the dynamics were truly due to the confinement of the cell mass, and not because the cells were bouncing against a “wall”. Therefore, instead of having a cell mass that pushes against a “wall”, we did simulations in which the cells were moving away from a “sticky wall”. In this case, we increased the *J_i,m_* values to prevent tissue fragments from breaking off and ending up in the medium. Such dynamics also lead to a pressure gradient, as in Figure 2, but in this case because volumes are larger than the target volumes. All results (i.e., the directional cell sorting along the pressure gradient and its specific dependency on the parameters) turn out to be equivalent to what we observed in the case of cell movement towards an undeformable wall (unpublished data).

## Discussion

We have shown that the speed and direction of movement of chemotactic cells in a sheet of tissue depends on their relative size and adhesion strengths, combined with properties of the tissue itself. In a confined cell mass, the chemotactic force in one direction creates a pressure gradient that generates a force in the opposite direction (Figure 4A). Differences in size or adhesion then cause differences in response to these two forces. Larger cells respond stronger to the pressure gradient than small cells, because they experience larger differences over their cell length. Consequently, cells with a larger volume move faster through a pressure gradient than smaller cells. They move backward, i.e., in the direction opposite to the chemotaxis, because the pressure gradient in the confined case is opposite to the chemotactic gradient (Figure 3; Video S1). Such dependence of movement speed on size has also been demonstrated, both experimentally and theoretically, in foams (which share certain basic mesoscale features with cells), for externally imposed pressure gradients [41]. Important for the process is the mesoscale structure of cells. By using a model formalism which not only explicitly takes cell structure into account, but also resolves forces that are exerted on the cell on a subcellular scale, it becomes apparent that forces act differently in different parts of the cell, causing cell deformation (Figure 4B and 4C), and eventually cell sorting.

The movement of cells that differ in adhesion properties depends on the “effective surface energy”, i.e., the surface energy relative to the cells' neighbours. In confined cell masses, if the average effective surface energy for one cell type is higher than that of the other cell type, the first cell type will sort forward (Figure 6; Videos S3–S8), because they better “resist” the pushing backward. Due to this mechanism, even cell types with negative surface tension can sort out (Figure 5D–5F), which is impossible by differential adhesion alone. Cell speeds and even direction of movement can change during the process: as cells form larger clusters, the effective surface energy changes, and clusters partly behave like larger-scale units, both influencing the sorting. The effective surface energy is also determined by the relative amounts of cells. If a cell mass consists of two cell types that only differ in cross-adhesion (i.e., *J_d,d_* = *J_l,l_* ≠ *J_d,l_*), the direction of cell sorting depends on the relative abundances, because the effective surface energy of the minority type is largely determined by the heterotypic bonds, and of the majority type by the homotypic bonds. We have called this “minority sorting”.

We have seen that cells can move against the direction of chemotaxis. Movement in the opposite direction of an imposed force has been termed, in physical systems, “absolute negative mobility”. It has been demonstrated in experiments and simulations of the motion of Brownian particles [42–45]. It is also observed in the well-known “Brazil nut problem”, the full understanding of which is still a source of debate [46]. Here we see that the negative mobility is caused by the mesoscale structure of cells, which persistently keeps the dynamics out of equilibrium. Using the same formalism (CPM), Zeng et al. [47] have presented an opposite view to cell sorting, in which the surface tension is initially negative, but during the sorting the homotypic bindings between the cells increase. Consequently, the dynamics “freeze” in a pattern with local phase separation, while preventing global sorting. In contrast, we have shown that to achieve fast global sorting, staying out of equilibrium is essential.

We applied these findings to study cell sorting in slime moulds. Cells of *Dictyostelium* move chemotactically towards the chemoattractant cAMP, which is produced and relayed in a wave-like fashion in the cell mass. The wave-like signal gives rise to wave-like chemotaxis and a complex pressure wave (Figure 8). This self-generated signal allows the cell mass to move forward as a “slug”. During this motion, so-called prestalk cells move to the front of the slug. As already pointed out [19,20], adhesion differences *(J_prestalk,prestalk_* < *J_prespore,prespore_)* alone, without chemotactic differences, suffice for cell sorting. Later, when the cell mass stops migrating, cells similar to prestalk cells (called anterior-like cells) move in the opposite direction to form the basal disk [48–50]. The results shown here may explain this change in direction of cell sorting solely on the basis of the transition from moving to attached cell mass, without assuming any differences between prestalk and anterior-like cells with respect to chemotaxis, adhesion, or size.

In this paper, we studied cell movement patterns assuming that cell properties are invariant. However, biological cells can dynamically change their properties. For example, just varying the number of adhesion molecules can be sufficient to generate differential adhesion [8]. The entanglement of cell differentiation and the processes described in this paper provide a very versatile substrate for morphogenesis [28,29]. Moreover, since the adhesion properties of a cell depend on the expression levels of cadherins [8], changing expression levels could be a possible way for directional cell motion and pattern formation to be modified during evolution. In ongoing work we are studying the morphogenesis of the slime mould *Polysphondylium,* which produces branched stalks, and comparing it with *Dictyostelium*. We try to find the minimum set of changes which could, via the dynamics of cell sorting studied in this paper, cause the strikingly different morphology of the fruiting body of these closely related species.

There are a number of ways in which these findings can be tested experimentally. First, the backward-directed pressure gradient can be verified by introducing a deformable object in cell tissues and tracking its speed depending on size. This could also give information about the forces cells are experiencing due to pressure gradients. Second, the minority sorting can be tested by constructing tissues with different relative abundances of the cell types. Third, the directional dependency on the confinement can be analysed by varying the viscosity of the medium; the slug stage of *Dictyostelium* would be an ideal subject. Finally, to test our predicted dependency of the direction and speed of sorting on the difference between homotypic and heterotypic adhesion, surface cadherin molecule expression experiments could be performed. Such experiments would also make it possible to move towards a more quantitative analysis of the interaction strengths involved in directional cell sorting.

In conclusion, we have shown that the combination of chemotaxis and differential adhesion by itself is a versatile mechanism for differential cell movement leading to predictable cell-sorting patterns within reasonable timescales. The behaviour of a cell depends not only on its own properties, but also on the characteristics of the tissue in which it finds itself. Physically driven cell rearrangements can profoundly influence the movement of biological cells (e.g., contra-chemotactic movement), and should not be neglected in explanations for observed cell movement patterns.

## Materials and Methods

For the simulations we use a 2-D square lattice consisting of 200 rows and (in most cases) 200 columns, with 1.3·10^3^ cells of two different cell types, i.e., dark *(d)* and light *(l)*. The cells are distributed randomly at the start of the run, 10% of them being dark. We use periodic boundary conditions to describe freely moving cell masses, or restrict movement at the border of the cell mass in the direction of chemotaxis to describe the dynamics of confined cell masses. In this study we explicitly focus on the role of cell–cell adhesion. We therefore use surface energies between cells and the medium that are high enough to prevent the cell mass from falling apart, but low enough to allow for rapid cell extensions into the medium (we use *J_l,m_* = *J_l,l_* and *J_d,m_* = *J_d,l_* – *J_l,m_* +1).

Constant chemotaxis is modelled by assuming a linear gradient in *c* (Equation 1), with slope 1/lattice site. To model cell sorting in *Dictyostelium,* we use the hybrid CPM/PDE model published earlier [20], which described the dynamics of the chemoattractant cAMP with a simplified FitzHugh–Nagumo model, with piecewise linear “Pushchino kinetics” [51]. Cells react chemotactically if the chemoattractant concentration is above a certain threshold and the refractoriness is below a threshold. Chemotaxis towards cAMP is described with Equation 1, substituting *c_site_* and c_*neighbour*_ with the computed cAMP concentrations.

## Supporting Information

Video S1 Size-Based Cell Sorting of a Cell Mass Moving Chemotactically towards the Right Parameters are as given in Figure 1, except for *V_d_* = 60 and *V_l_* = 30. The total length of the movie is 240,000 MCS.
(3.3 MB MPG)

Video S2 Size-Based Cell Sorting without Chemotaxis, but due to a Pressure Gradient That Is Created by Pushing the Cells Forward, Towards the Right Parameters are the same as for Video S1, except for *T* = 1 and *μ* = 0. The total length of the movie is 33,000 MCS.
(3.4 MB MPG)

Video S3 Cell Sorting due to Differential Adhesion of a Cell Mass Moving Chemotactically towards the Right Videos S3–S8 show the dynamics for each qualitatively different region in Figure 6. Video S3 shows the dynamics in region (A). *J_l,l_* = 5, *J_d,l_* = 3, and *J_d,d_* = 5. All other parameters are as given in Figure 1. The total length of the movie is 10^5^ MCS.
(2.7 MB MPG)

Video S4 Dynamics in Region (B) of Figure 6
Parameters are the same as for Video S3, except for *J_d,l_* = 6, *J_d,d_* = 9. The total length of the movie is 10^6^ MCS.
(1.9 MB MPG)

Video S5 Dynamics in Region (C) of Figure 6
Parameters are the same as for Video S3, except for *J_d,l_* = 4, *J_d,d_* = 2. The total length of the movie is 7·10^5^ MCS.
(2.7 MB MPG)

Video S6 Dynamics in Region (D) of Figure 6
Parameters are the same as for Video S3, except for *J_d,l_* = 8, *J_d,d_* = 2. The total length of the movie is 10^6^ MCS.
(2.2 MB MPG)

Video S7 Dynamics in Region (E) of Figure 6
Parameters are the same as for Video S3, except for *J_d,l_* = 9, *J_d,d_* = 7. The total length of the movie is 10^6^ MCS.
(2.7 MB MPG)

Video S8 Dynamics in Region (F) of Figure 6
Parameters are the same as for Video S3, except for *J_d,l_* = 8, *J_d,d_* = 9. The total length of the movie is 6.7·10^5^ MCS.
(1.8 MB MPG)

## **Abbreviations:**

CPM: Cellular Potts Model
MCS: Monte Carlo time step
